# Brain cancer patients' levels of distress and supportive care needs over time

**DOI:** 10.1002/pon.6028

**Published:** 2022-09-27

**Authors:** Georgia K. B. Halkett, Elizabeth Lobb, Katrina Spilsbury, Haryana Dhillon, Anna K. Nowak

**Affiliations:** ^1^ Curtin School of Nursing Faculty of Health Sciences Perth Western Australia Australia; ^2^ Curtin Health Innovation Research Institute (CHIRI) Curtin University Bentley Western Australia Australia; ^3^ Calvary Health Care Kogarah Sydney New South Wales Australia; ^4^ School of Medicine The University of Notre Dame Sydney New South Wales Australia; ^5^ Faculty of Health University of Technology Sydney Ultimo New South Wales Australia; ^6^ Institute for Health Research University of Notre Dame Australia Fremantle Western Australia Australia; ^7^ Psycho‐Oncology Cooperative Research Group School of Psychology Faculty of Science University of Sydney Sydney New South Wales Australia; ^8^ Centre for Medical Psychology & Evidence‐based Decision‐making School of Psychology Faculty of Science University of Sydney Sydney New South Wales Australia; ^9^ Medical School University of Western Australia Nedlands Western Australia Australia; ^10^ Department of Medical Oncology Sir Charles Gairdner Hospital Nedlands Western Australia Australia

**Keywords:** brain tumours, high grade glioma, longitudinal study, patient distress, supportive care needs

## Abstract

**Purpose:**

This study aimed to describe patient self‐reported distress over time and how this was associated with wellbeing, and supportive care needs over a 6‐month period from commencing chemoradiotherapy for high grade glioma (HGG).

**Methods:**

In this prospective cohort study, participants completed surveys at three time points: before chemoradiotherapy, at 3 and 6 months. These included Distress Thermometer, Functional Assessment of Cancer/Brain Cancer Treatment‐general (Fact‐G/FACT‐BR), Supportive Care Needs Scale (SF‐34) and Brain Tumour Specific subscale. Patient survival time was also collected. Group‐based trajectory modelling was performed. Multinominal logistic regression assessed variables associated with different distress trajectory groups.

**Results:**

One hundred and sixteen participants completed assessments at baseline, 89 participants at 3 and 64 at 6 months. Four distress trajectory groups were identified; consistent low distress (18%), low to high distress (38%), high‐to low distress (24%) and consistent high distress (19%). Younger participants tended to report decreased distress over time, whereas older participants reported consistently high distress. High distress trajectory participants had less education, lower physical wellbeing, more unmet needs, but higher functional wellbeing compared to the low to high distress trajectory. The number of unmet needs paralleled the patterns of distress over time. The highest unmet needs in people with HGG and high distress were disease specific changes in mental ability and physical side effects.

**Conclusion:**

This study demonstrates people with HGG experience ongoing distress and highlights a need for continuous distress and unmet needs screening and referrals.

## 1 BACKGROUND

People diagnosed with High Grade Glioma (HGG) (Grade III‐IV Glioma) experience functional, emotional, and cognitive decline.^1^, ^2^ Median survival for patients who receive treatment is around 15 months for Grade IV^3^ and 5 years for Grade III glioma.^4^ People with HGG and their carers must manage emotions, make treatment decisions, and rapidly adjust to comprehensive lifestyle changes.^5^, ^6^, ^7^ Previous studies have reported up to 50% of people with brain tumours experience high levels of distress,^8^ anxiety, and depression.^9^, ^10^ The diagnosis is frequently associated with an inability to work, drive, or participate in previous activities.^11^, ^12^ A recent systematic review^13^ identified that patients with primary brain tumours may experience existential distress (fear of recurrence/progression and dying) which correlated with higher levels of depression and anxiety, and poorer quality of life (QOL).

Previous research indicates people with HGG experience significantly lower QOL than population norms.^14^, ^15^ QOL for HGG patients can be impacted by tumour type and location, treatment received, gender, neurocognitive functioning, mood disorders, medications prescribed for symptoms and comorbidities (e.g. steroids, antiepileptics and analgesics), and fatigue and sleep problems.^16^, ^17^ The anxiety and depression experienced is associated with poorer QOL.^14^, ^15^ High levels of distress, poor QOL, and a poorer clinical condition is linked to unmet needs including dissatisfaction with information and support from health professionals.^9^, ^10^ Unmet needs for people with HGG have been reported as information about disease progression, treatment, psychological and social support, communication with health professionals, care coordination, dealing with fatigue, uncertainty about the future, difficulty with activities of daily living, and access to support services.^14^, ^15^, ^18^, ^19^ Distress is consistently associated with unmet supportive care needs in the psychological domain for people with HGG at different stages of treatment.^14^

In a longitudinal mixed method study in 30 people with HGG, mean anxiety was highest at baseline and decreased over time and emotional well‐being increased significantly from baseline.^1^ Anxiety was related to overall QOL and emotional well‐being. Depression was associated with poorer QOL. Following this study, Piil et al.^20^ conducted a longitudinal mixed‐methods study with 30 patients with glioma and 33 carers identifying that patients' needs change over time depending on disease progression and individual preferences. Piil et al highlighted the need for additional supportive care (including screening for depression and facilitating referral to effective interventions), education and information (including information about disease trajectory and prognosis and managing functional and daily adjustments, health promotion activities and complementary therapies and palliative care) and rehabilitation (interventions for cognitive and physical function). Further longitudinal research is required to explore the link between QOL, distress and unmet needs over time in people with HGG.

We previously reported people diagnosed with HGG (*n* = 116) had a poor QOL, increased distress and high levels of unmet need at the commencement of combined chemoradiation compared to the general population.^21^ Poorer physical function, lower education levels, loss of employment, and financial impact were linked with multiple domains of distress, poorer QOL and high unmet needs.^21^ We have previously published data for carers of people with HGG highlighting carers remain highly distressed over time and have changing unmet needs as the patients' disease progresses.^22^, ^23^ Additionally, we found higher distress levels were associated cross‐sectionally with carers having a higher number of unmet needs.^22^

In the current study we aimed to describe variation in patient self‐reported distress and how this was associated with wellbeing and unmet supportive care needs over a 6‐month period from start of combined chemoradiotherapy for World Health Organisation (WHO) Grade III—IV HGG. Hypotheses: (1) Participants experience increasing distress from commencement of chemoradiotherapy until 6 months later as their disease progresses; and, (2) High distress is associated with higher unmet needs at baseline, 3 and 6 months later.

## 2 METHODS

### 2.1 Study design

We conducted a prospective cohort study. Detailed information on participant recruitment, study tools, and baseline outcomes have been reported previously.^21^ In brief, potential participants were recruited from neurosurgical, radiation, or medical oncology outpatient clinics from four tertiary hospitals in two Australian states. Treating clinicians or the cancer nurse coordinator recruited participants into the study. Eligible participants provided informed consent, were aged over 18 years and planned to begin chemoradiotherapy treatment for HGG. Potential participants were excluded if they were unable to complete questionnaires for language, literacy, or medical reasons. Participants and carers completed questionnaires (in the clinic or at home and returned via mail) at three time points: during chemoradiotherapy and 3 and 6 months later. Approval to conduct this study was obtained from Human Research Ethics Committees at each site.

### 2.2 Participant questionnaires

The questionnaire comprised four sections. Section 1 collected socio‐demographic information including details of carers (if any), financial burden of diagnosis, and patient self‐report of level of physical function based on the Eastern Co‐operative Oncology Group (ECOG) performance status ranging from 0 (fully independent) to 4 (completely disabled). Section 2 contained the Distress Thermometer (DT) a single item 11‐point Likert‐like scale ranging from 0 (no distress) to 10 (extreme distress).^24^ Section 3 comprised questions relating to QOL from the Functional Assessment of Cancer Therapy‐general (FACT‐G) assessing wellbeing through physical, social, emotional and functional subscales.^25^ A validated brain cancer specific subscale was also included.^26^ Section 4 included items from the Supportive Care Needs Scale (SCNS) tools designed to identify how well patients feel their specific cancer‐related needs are being met. We used the SCNS‐SF34, a 34‐item scale related to psychological, physical and daily living, health system and information, sexuality and patient support^27^; the 16‐items related to service access needs; and the 16‐item brain tumour specific tool.^14^

Survival time was defined as time from date of diagnosis to date of death with patients censored at date last known alive (if lost to follow up) or study censor date (31 December 2017) for those patients known to be alive. Proximity to death was defined as the time in months between date of death and date of each survey. This was censored at 50 months for the 5% who survived past 50 months or were alive at study censor date.

Self‐reported carer distress measured using the DT at each of the three time points was included as a covariate in this analysis. All other carer data is reported elsewhere.^22^, ^23^

### 2.3 Data analysis

Equality of means and proportions were assessed using *t*‐tests and chi‐square tests respectively.

The DT is traditionally analysed by defining a cut‐point representing distress. The original cut‐off point used was 5 (midpoint of 11‐point scale), however, more recent studies propose 4 as cut‐off points for identifying people with HGG with clinically meaningful distress, although this can vary by diagnosis. We used a cut point of 4 and above to identify ‘moderate’ distress, and a cut point of 7 and above to represent ‘severe’ distress.^28^ Both the SCNS and FACT‐G responses were converted to a standardised (prorated) Likert summated score over subscales as recommended.^29^

Distress has been reported to be higher close to time of diagnosis,^8^ so we used time since diagnosis as the timescale for the longitudinal analyses. We used the date of questionnaire return to indicate participant survey completion date.

Group‐based trajectory modelling was used to assign probabilities of individual patients following different trajectories of distress over time.^30^ Trajectory groups themselves are a convenient statistical tool and are not necessarily interpreted at the individual patient level. Missing data due to death or withdrawal was accommodated by directly modelling a constant attrition process and allowing it to vary across trajectory groups. A balance between maximising the Bayesian information criterion (BIC), having an average posterior probability >0.7 for each group, having sufficient numbers per group, and clinical plausibility was used for trajectory model selection. Baseline sociodemographic characteristics, carer distress, closeness to time of death, and patient reported wellbeing associated with trajectory groups were assessed using multinomial logistic regression weighted by patient probability of trajectory group membership. The Kaplan‐Meier product limit estimate of the survivor function was used to obtain median survival times with associated standard errors and confidence intervals estimated based on the restricted mean method. Data analyses were performed using Stata v16 (College Station).

## 3 RESULTS

One hundred and sixteen participants completed the baseline survey. The sociodemographic and clinical characteristics of the cohort and baseline measures of distress and QOL have been reported elsewhere.^16^ In brief, 71% were male, 94% had grade IV glioma, most (83%) had a current partner, 52% were tertiary educated, and 94% reported having a carer at time of enrolment. The mean age at baseline of the 116 participants was 55.7 years (SD 13.0; range 18–86). Forty‐two percent of participants reported their diagnosis had a significant effect on their financial circumstances.

**FIGURE 1:**
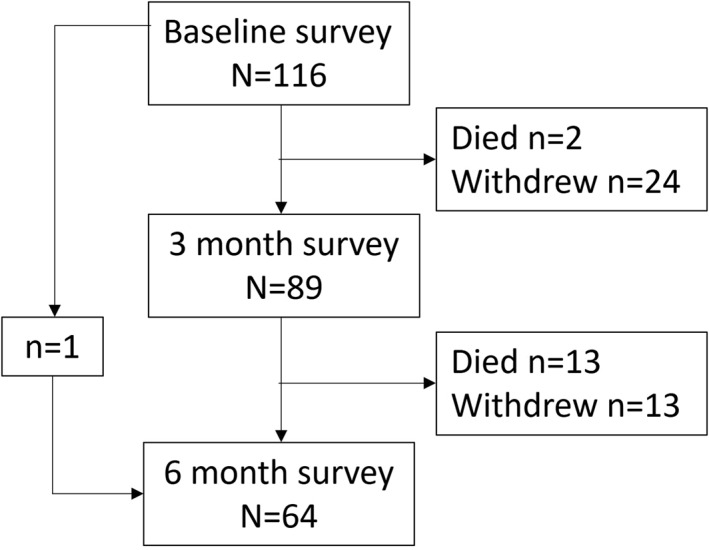
Study participation

Eighty‐nine participants completed the survey at 3 and 64 at 6 months. Baseline characteristics, distress levels, wellbeing and supportive care needs of the 64 participants at 6 months were compared with the 52 participants who died or withdrew from the study after completing the baseline questionnaire to assess differences between groups (Figure 1 and Table 1). Participant groups differed only in that those lost to follow‐up were significantly closer to time of death and reported slightly higher emotional wellbeing at baseline. Supplement 1 provides survival curve of patients stratified by whether they completed all three surveys or not.

**TABLE 1:** Baseline characteristics (time of first survey completion) of participants who completed the study and those lost to follow‐up

	Completed study		Lost to follow‐up		*p*‐value
	*N* = 64		*N* = 52		
	Mean	SD	Mean	SD	
Age (years)	54.4	12.6	57.2	13.6	0.248
Distress thermometer	4.2	2.9	4.0	2.9	0.694
Carer distress thermometer	5.0	2.7	5.3	2.4	0.571
Median survival time months (95% CI)^a^	20.1	17.0–23.3	11.2	8.4–12.7	<0.001*
FACT domain scores					
Emotional wellbeing	15.8	5.7	17.9	5.0	0.036*
Social wellbeing	23.5	4.3	22.8	5.2	0.390
Physical wellbeing	21.7	4.4	19.8	6.7	0.073
Functional wellbeing	16.0	6.2	15.1	5.9	0.425
Total general wellbeing	77.1	14.3	75.8	17.0	0.658
Brain cancer specific	49.5	12.9	47.5	12.7	0.403
Total brain cancer	126.3	24.1	123.5	26.5	0.552

	***N***	**%**	***N***	**%**	
Sex					
Male	48	75.0	34	65.4	0.258
Female	16	25.0	18	34.6	
Partnered					
No	10	15.6	11	21.2	0.442
Yes	54	84.4	41	78.8	
Tertiary education					
No	30	46.9	26	50.0	0.738
Yes	34	53.1	26	50.0	
Employment status before diagnosis					
Not employed	27	42.2	23	44.2	0.825
Employed	37	57.8	29	55.8	
Financial impact of diagnosis					
No or slight effect	39	60.9	25	51.0	0.292
Significant effect	25	39.1	24	49.0	
Level of dependency—ECOG					
Independent	23	35.9	14	26.9	0.434
Restricted	21	32.8	20	38.5	
More restricted	15	23.4	12	23.1	
Very restricted	5	7.8	3	5.8	
Completely restricted	0	0.0	2	3.8	
Mobility aids used					
None	55	85.9	43	82.7	0.870
Walking stick	4	6.3	4	7.7	
Walking frame	2	3.1	1	1.9	
Wheelchair	3	4.7	4	7.7	

^a^ Smaller number for median survival indicates patients in the lost to follow‐up group died sooner (median survival time was shorter).

*Significant scores are presented in bold with an asterisk.

### 3.1 Temporal change in distress

**FIGURE 2:**
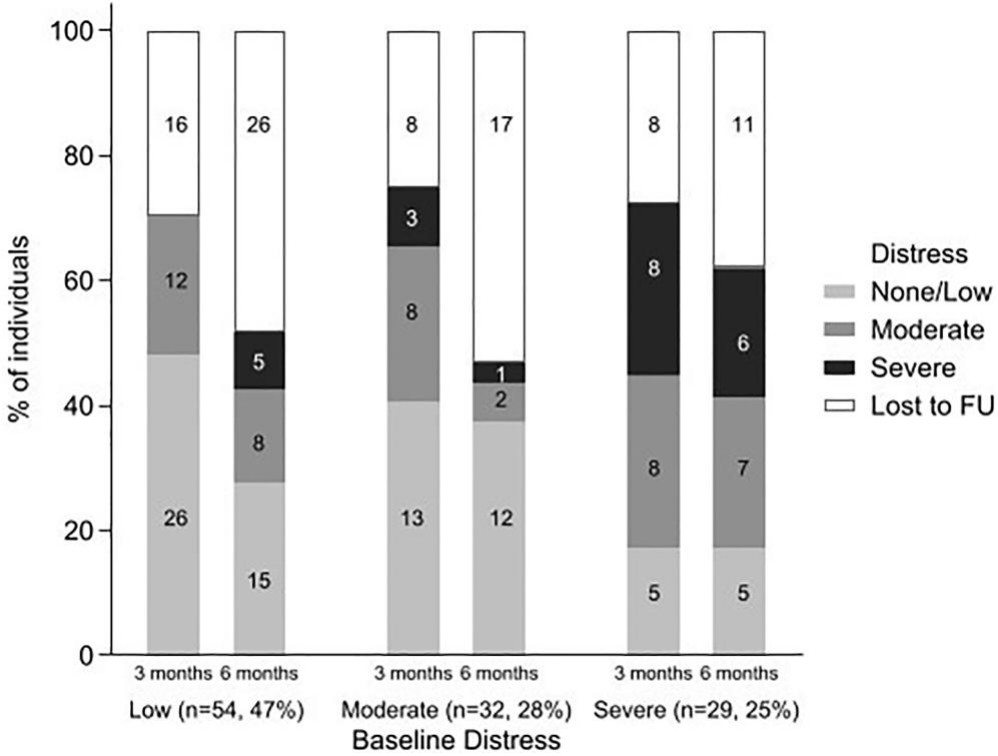
Number and percentage of participants reporting low, moderate, or severe distress at three and 6 months stratified by baseline distress category (start of chemoradiotherapy)

Individual patterns of participant distress levels were highly variable over time (baseline, 3 and 6 months) (Figure 2). Some reported high distress at baseline, but lower distress levels at 3 and 6 months. Others reported no distress at baseline, but increasing distress, while yet others reported more stable levels of distress over time.

Of participants who reported no distress at baseline, around 45% remaining in the study reported moderate or severe distress at later time points. Whereas of those reporting severe distress at baseline, almost one third those who remained in the study reported no distress at later time periods.

Group‐based trajectory analyses was performed to identify other factors associated with groups of participants who tended to follow similar patterns of distress over time, while accounting for patterns of attrition.^31^ Four trajectory groups were identified; low distress (18%), low to high distress (38%), high‐to low distress (24%) and high distress (19%) (Supplement 2). Participant attrition rates were similar in all four trajectory groups at around 16%–18% per time point.

### 3.2 Participant baseline differences by distress trajectory groups

Of all participants in distress trajectory groups who started with high distress, younger participants tended to report decreased distress over time, whereas those reporting consistently high distress were older on average (Table 2). Baseline wellbeing measures also varied between distress trajectory groups. The high distress trajectory group had the lowest mean wellbeing scores in the physical, functional, and emotional domains and overall. The low distress trajectory group reported the greatest physical, functional, and emotional wellbeing. Baseline social wellbeing did not vary between groups.

**TABLE 2:** Characteristics reported at baseline stratified by distress trajectory group

	Distress trajectory group									
	Low (*n* = 19)		Low to high (*n* = 52)		High to low (*n* = 21)		High (*n* = 24)		*p* value	
	Mean	SD	Mean	SD	Mean	SD	Mean	SD		
Age (years)	56.6	14.2	56.8	12.4	47.6	15.2	59.5	8.5	0.012*
FACT wellbeing									
Social	24.9	3.4	23.0	5.6	22.6	3.9	23.0	4.1	0.374
Functional	21.7	5.1	15.5	5.6	14.5	4.6	12.0	5.1	<0.001*
Emotional	21.4	3.2	17.3	4.7	14.4	5.7	13.8	5.4	<0.001*
Physical	24.9	2.7	21.4	5.2	20.5	4.8	16.4	6.0	<0.001*
Total ‐general	93.0	8.8	77.2	15.1	72.0	12.5	65.2	10.9	<0.001*
Brain cancer specific	57.4	10.5	49.4	11.9	50.8	11.0	37.8	11.1	<0.001*
Total—general + Br cancer	150.5	16.9	126.6	23.5	122.1	18.2	103.1	19.4	<0.001*
Carer baseline distress	4.4	3.1	4.7	2.2	5.5	2.8	6.3	2.2	0.031*
No. Unmet needs (SCNS)	6.5	8.4	17.4	13.7	24.0	13.6	29.5	15.6	<0.001*
Median survival time (SE)	19.9	2.8	13.3	1.7	21.2	13.5	14.6	2.6	0.010*

	***N***	**%**	***N***	**%**	***N***	**%**	***N***	**%**	
Sex									
Male	15	78.9	33	63.5	17	81.0	17	70.8	0.391
Female	4	21.1	19	36.5	4	19.0	7	29.2	
Partnered									
No	5	26.3	11	21.2	2	9.5	3	12.5	0.433
Yes	14	73.7	41	78.8	19	90.5	21	87.5	
Tertiary education									
No	8	42.1	17	32.7	13	61.9	18	75.0	0.003*
Yes	11	57.9	35	67.3	8	38.1	6	25.0	
Financial impact of diagnosis									
No or slight effect	14	73.7	30	60.0	11	52.4	9	39.1	0.138
Significant effect	5	26.3	20	40.0	10	47.6	14	60.9	
Carer type									
Partner/spouse	13	68.4	40	76.9	18	85.7	21	87.5	0.593
Other carers	3	15.8	9	17.3	2	9.5	2	8.3	
None	3	15.8	3	5.8	1	4.8	1	4.2	
Employment status									
Not employed	11	57.9	33	63.5	14	66.7	17	70.8	0.748
Full‐time employed	7	36.8	14	26.9	5	23.8	7	29.2	
Part‐time employed	1	5.3	5	9.6	2	9.5	0	0.0	
Mobility aids used									
None	18	94.7	40	76.9	20	95.2	20	83.3	0.392
Walking stick	0	0.0	5	9.6	1	4.8	2	8.3	
Walking frame	1	5.3	1	1.9	0	0.0	1	4.2	
Wheelchair	0	0.0	6	11.5	0	0.0	1	4.2	
Level of dependency									
Independent	12	63.2	15	28.8	6	28.6	4	16.7	0.027*
Restricted	4	21.1	17	32.7	10	47.6	10	41.7	
More restricted	2	10.5	15	28.8	3	14.3	7	29.2	
Very restricted	1	5.3	5	9.6	2	9.5	0	0.0	
Completely restricted	0	0.0	0	0.0	0	0.0	2	8.3	
Unknown	0	0.0	0	0.0	0	0.0	1	4.2	
Felt involved in decision process									
No	4	21.1	7	13.5	6	28.6	3	12.5	0.394
Yes	15	78.9	45	86.5	15	71.4	21	87.5	
High difficulty understanding									
No	15	78.9	49	94.2	18	85.7	19	79.2	0.183
Yes	4	21.1	3	5.8	3	14.3	5	20.8	

Abbreviations: SD, standard deviation; SE, standard error.

*Significant scores are presented in bold with an asterisk.

The high distress trajectory group of participants reported a much greater number of unmet needs at time of chemoradiotherapy and evidence their carers were more distressed at baseline (Table 2). This group comprised significantly fewer patients with a tertiary education than the other groups. Participants in the high and the low to high distress trajectory groups had shorter survival times and reported more baseline dependency than the other distress trajectory groups.

A multinomial logistic regression model was constructed to determine which of these baseline characteristics remained associated with distress trajectory group membership after adjusting for other variables (Table 3). Relative to participants with high probability of being in the low to high distress group, those with constant low distress reported higher functional wellbeing and tended to live longer. High distress trajectory participants had less education, lower physical wellbeing, more unmet needs, but higher functional wellbeing compared to the low to high distress trajectory. Participants in the high to low distress trajectory tended to have longer survival, less education, and more unmet needs than the low to high distress trajectory groups.

**TABLE 3:** Multivariable modelled risk ratios of reported baseline characteristics associated with distress trajectory group membership relative to participants in the low to high distress trajectory group

Baseline characteristics	RRR	95% CI	*p*‐value
Low distress			
Physical wellbeing	1.32	0.95–1.85	0.103
Functional wellbeing	1.19	1.02–1.40	0.030*
Tertiary education	0.27	0.06–1.26	0.095
Survival time	1.05	1.00–1.10	0.072
Total unmet needs	0.95	0.86–1.04	0.275
High to low distress			
Physical wellbeing	1.02	0.91–1.14	0.769
Functional wellbeing	1.01	0.89–1.15	0.843
Tertiary education	0.29	0.09–0.98	0.046*
Survival time	1.06	1.01–1.10	0.008*
Total unmet needs	1.05	1.00–1.10	0.033*
High distress			
Physical wellbeing	0.80	0.70–0.92	0.002*
Functional wellbeing	1.16	1.03–1.31	0.015*
Tertiary education	0.10	0.02–0.50	0.005*
Survival time	1.01	0.96–1.05	0.703
Total unmet needs	1.06	1.01–1.12	0.011*

*Significant scores are presented in bold with an asterisk.

### 3.3 Distress trajectory groups and unmet needs

The baseline number of unmet needs was higher in the distress trajectory groups that started high from the time of first survey (Table 3). A summary of the number of unmet needs over time is shown in Supplement 3. Broadly speaking, the number of unmet needs paralleled the patterns of distress over time.

**FIGURE 3:**
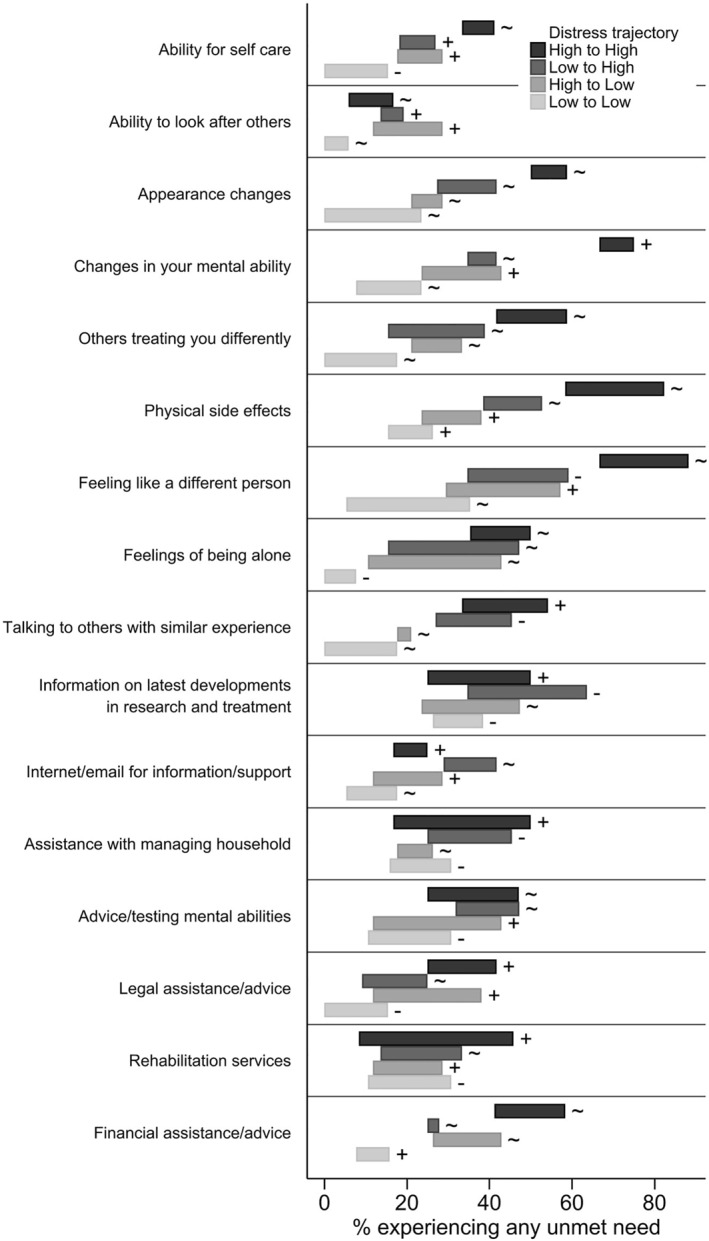
Range of the proportion of participants experiencing any brain cancer specific unmet needs from baseline to 6 months by distress trajectory group with indication of consistent increase (+), consistent decrease (−), or fluctuating proportions (∼) over time

**FIGURE 4:**
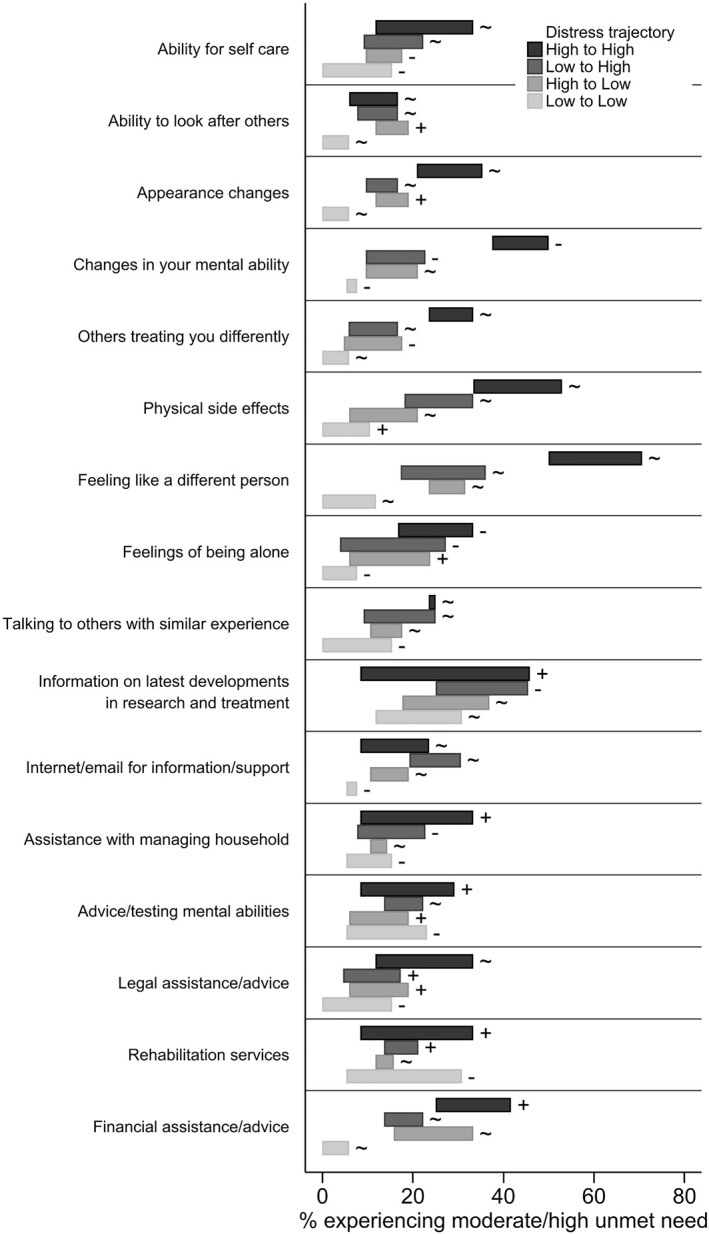
Range of the proportion of participants experiencing moderate to high brain cancer specific unmet needs from baseline to 6 months by distress trajectory group with indication of consistent increase (+), consistent decrease (−), or fluctuating proportions (∼) over time

With focus on the 16 questions of the brain cancer‐specific module of unmet needs, we observed distinct patterns of unmet needs by distress trajectory group. We assessed both any unmet need (low/moderate/high) (Figure 3) and moderate/high unmet needs only (Figure 4). The highest unmet needs relating to brain cancer in people with high distress included: changes in mental ability, physical side effects, feeling like a different person, appearance changes, and others treating them differently. While these needs decreased or fluctuated, they continued to remain higher in the high distress group compared to other groups. Legal assistance was a high need which fluctuated over time for participants in the high distress group. Those in the high distress group also had an increasing need for financial assistance/advice, information on latest development in research and treatment, assistance with managing household, accessing rehabilitation services, and advice/testing mental abilities. Supplement 4 shows the range of the proportion of patients in each distress trajectory group reporting unmet needs for all 66 questions in the SNSC survey over the three survey time points.

## 4 DISCUSSION

This study aimed to describe individuals' self‐reported distress and how this related to wellbeing and supportive care needs over a 6 month period from starting combined chemoradiotherapy for HGG. While we initially hypothesised participants would experience increasing distress over time, our findings demonstrated a variety of distress trajectories. Group‐based trajectory modelling revealed some people experienced persistent distress throughout the study, while others maintained low levels of distress and others moved from high to low or vice‐versa. Individuals may have different coping skills and their response to their diagnosis depends on support received and how others are coping with their disease.

Importantly, high distress at the time of starting chemoradiotherapy reflected a higher number of unmet needs, demonstrating the strong relationship between distress and unmet needs as seen in other populations during active treatment^32^, ^33^ through to survivorship.^34^ ^,^ ^35^ Regular monitoring of patient distress is necessary as distress was prevalent at all three time points. Lack of distress at an earlier timepoint did not preclude development of high distress levels. We previously reported the importance of ongoing screening for distress in carers of people diagnosed with HGG^22^, ^23^ and in our earlier qualitative research with patients and carers.^36^ Additionally, supportive care interventions and resources are required to ensure health services are able to respond effectively when patients and carers have high distress scores.

We found higher patient distress was associated with higher carer baseline distress and a greater number of unmet needs when chemoradiotherapy commenced. Our results identified practical, physical, and emotional unmet needs in these populations that are clearly driving distress. Most of this unmet need centred on the unwanted physical and mental changes specific to brain cancer. While needs decreased or fluctuated they remained higher in the high distress group compared to others. Legal assistance (from the brain tumour specific needs tool) was a high need which fluctuated over time for participants in the high distress group. The high distress group also identified, on the brain tumour specific needs tool, that they had increasing needs for financial assistance/advice, information on new research and treatment options, practical support at home, accessing rehabilitation services and neuropsychological support.

The brain tumour specific unmet needs identified and associated with higher distress highlight the urgent need for routine screening for unmet needs, increased referral to support services, and improved interventions to address these needs for the wellbeing of patients and carers. Renovanz et al.^10^ highlighted the crucial role clinicians play in supporting people with brain tumours and proposed clinicians routinely ask questions about distress during consultations to identify problems experienced and initiate support where required. Fortunato et al.^37^ also highlighted the importance of communicating with carers to improve end of life care and reduce carer distress burnout and bereavement. Targeted communication skills training for clinicians focusing on communicating and assessing distress and unmet needs for people diagnosed with a brain tumour and their carers may assist clinicians in providing support and referring appropriately.^38^ It is imperative cancer services, primary health care networks, and non‐government organisations such as Cancer Councils, work together to fill the gaps in care. Further research is warranted to trial interventions to reduce patient and carer distress and address brain cancer specific unmet needs.

Of all patients in the different distress trajectory groups who started with high distress, younger participants tended to report decreased distress over time, whereas those reporting consistently high distress were older on average. Previous research has highlighted the need to identify at risk adolescent and young adult cancer patients and refer them for psychosocial support.^39^ Health professionals working with younger patients may be primed to assess distress and unmet needs in younger patients due to their life circumstances. However, distress and unmet needs must also be assessed in older cancer patients.^40^, ^41^ The higher levels of distress older participants experienced in our study presents a strong argument for routine distress screening to ensure those in need of support and intervention are recognised and assisted.

Participants in the high distress and the low to high distress trajectory groups had shorter survival times and reported more baseline dependency than the other groups reflecting both deteriorating health states and recognition of limited remaining life. Higher distress for participants with shorter survival times is unsurprising with this group facing their own mortality and likely to be experiencing progressive neurological deficits, although we did not specifically assess for this. Recent work by Loughan et al.^42^ with 105 patients found people with primary brain tumours experience a high prevalence of death‐related distress with death anxiety being endorsed by 81% of participants. In the current study we found participants with higher distress reported higher needs at 6 months related to feelings about death and dying, uncertainty about the future, and keeping a positive outlook (see Supplement 4). Our findings suggest early communication about prognosis, disease trajectory, and support to plan for end of life may help adjustment to the disease and, particularly, carer coping.

Interventions focusing on supporting people with HGG and carers to identify their fears and plan for the future such as Tele‐MAST (a telehealth delivered psychotherapeutic intervention for people with primary brain tumour and family members to support them to ‘MAke Sense of brain Tumours (MAST)’) may be beneficial in reducing patient depression and improving patient and carer mental health and QOL.^43^, ^44^ Previously, Ownsworth et al.^45^ evaluated a home‐based psychosocial intervention (10 1 hour sessions) for patients (*n* = 50) finding that at 6 months participants had lower levels of depression and stress and higher existential well‐being and QOL. Our team has also been trialling a nurse‐led intervention (Care‐IS) with carers of HGG patients to improve carer preparedness and reduce carer distress.^46^ Piil et al.^47^ recommended that emotional support provided by health professionals requires improvement and should include family members. Further research is required to trial supportive care interventions for patients and their carers following a diagnosis of HGG.

### 4.1 Clinical implications

Our data demonstrate the urgent need for policy changes to implement regular screening for distress and unmet needs in HGG populations. To enable needs of patients and carers to be effectively addressed, clinical pathways to guide referrals, increased workforce particularly neuropsychology, and targeted interventions are critical. We have successfully implemented a stepped‐care model for management of distress, anxiety, and depression in general cancer populations incorporating psycho‐education, online cognitive behaviour therapy, and referral for in person care.^48^, ^49^ Establishing such clinical pathways in people with brain tumours, working together across health jurisdictions and the community sector is the best way to fill the gaps in care.

Systematically screening and providing support flexibly may help overcome disparities in access to support services in vulnerable HGG populations. Technological solutions may facilitate screening and access to interventions and resources. Of particular importance, is the need to assess the effectiveness of telehealth/internet delivered services to increase access in people with limited mobility, time, financial resources, and a high burden of health system interactions.

It is clear advanced care and end‐of‐life planning should be discussed with patients and families early. Our data depicts high levels of distress and existential concerns as end‐of‐life approaches. What is unknown is how often and how effectively these conversations occur. Advanced and end‐of‐life care planning should not be a one‐time conversation, but continued throughout the care trajectory to assess and assist with changing needs and perceptions.

### 4.2 Limitations

We conducted this exploratory study over a 6‐month period, but acknowledge distress and unmet needs are likely to be affected by key clinical events, such as disease recurrence, which we did not assess. The study time points were selected to balance the information obtained with participant burden. Participants had the choice to either complete the questionnaires in the clinic or at home and carers were able to assist with the completion of the surveys. While this is a limitation of the study, involvement of carers increased completion rates and assisted with patient comprehension. Completion rates declined over time, as anticipated in a group juggling competing demands and declining health. However, participant withdrawal was accounted for during trajectory analysis and participant characteristics did not differ between those who continued or withdrew due to disease progression. Additional data collection after 6 months would have provided further insight into participant distress and needs; however, we were cognisant of the difficulties of maintaining adherence and chose to stop at 6 months to reduce participant burden and account for the high attrition due to deterioration in patient health and the associated increase in caregiver burden.

## 5 CONCLUSION

This study has demonstrated people continue to experience distress following a diagnosis of HGG and are likely to benefit from screening for distress and unmet needs. People need support dealing with unmet needs specific to brain cancer including: changes in mental ability, physical side effects, feeling like a different person, appearance changes and others treating them differently. Interventions should include support for both patients and carers and must focus on addressing these unmet needs to reduce distress.

## AUTHOR CONTRIBUTIONS

Georgia Halkett, Elizabeth Lobb and Anna Nowak contributed to the study conception and design. Katrina Spilsbury provided statistical advice and analysed the data. Critical feedback on data analysis and interpretation was provided by all authors. The first draft of the manuscript was written by Georgia Halkett and all authors commented on multiple versions of the manuscript. All authors read and approved the final manuscript.

## CONFLICTS OF INTEREST

The authors have no relevant financial or non‐financial interests to disclose.

## ETHICS STATEMENT

This study was performed in line with the principles of the Declaration of Helsinki. Approval was granted by the Human Research Ethics Committees of Sir Charles Gairdner Hospital (2006‐146), Curtin University (03/2007), and Cancer Institute NSW (2008/08/092).

## CONSENT TO PARTICIPATE

Witten informed consent was obtained from all individual participants included in the study.

## Supporting information

Supporting Information S1

## Data Availability

The datasets generated during and/or analysed during the current study are available from the corresponding author on reasonable request.
