# Supplementary material for: Brain cancer patients' levels of distress and supportive care needs over time
Source: Psychooncology. 2022 Sep 27;31(12):2074–85. doi: 10.1002/pon.6028 (PMC10087040; doi:10.1002/pon.6028)
Supplement: Supplementary file 1 — Supporting Information S1 [file PON-31-2074-s001.docx]

**Supplementary Files**

Supplement 1: Survival curve of patients stratified by whether they completed all three surveys or not.

**

Supplement 2: A) Four predicted trajectory groups (solid lines and average distress scores per group plotted (marker points) B) Individual patient distress scores over time stratified by trajectory group.


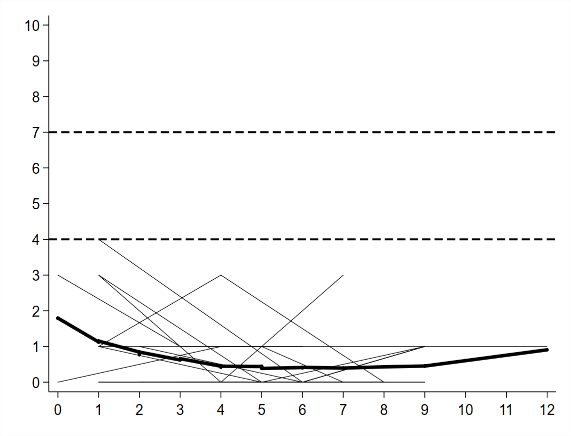

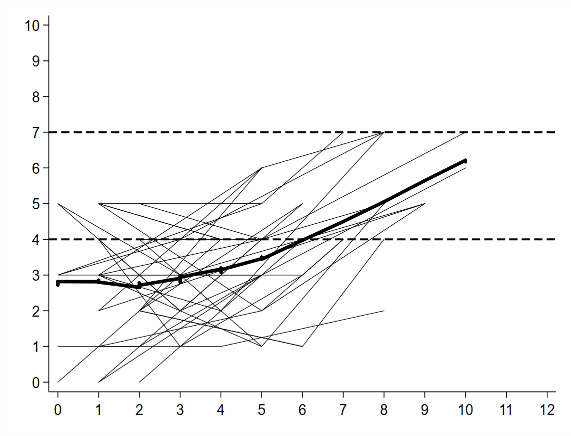

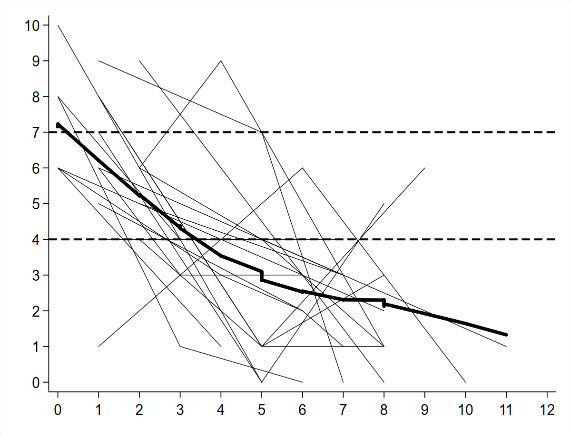

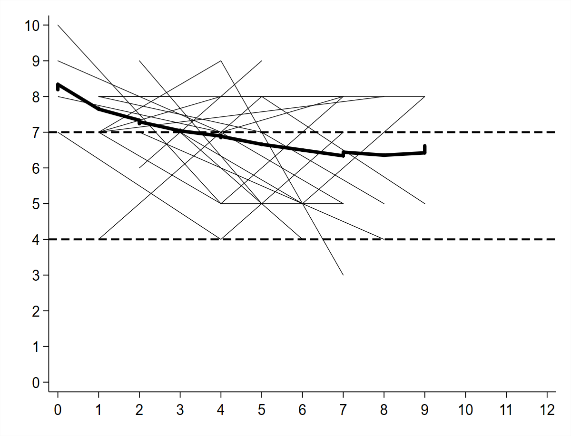


Months since diagnosis

Distress thermometer

Low

Low to high

High to low

High


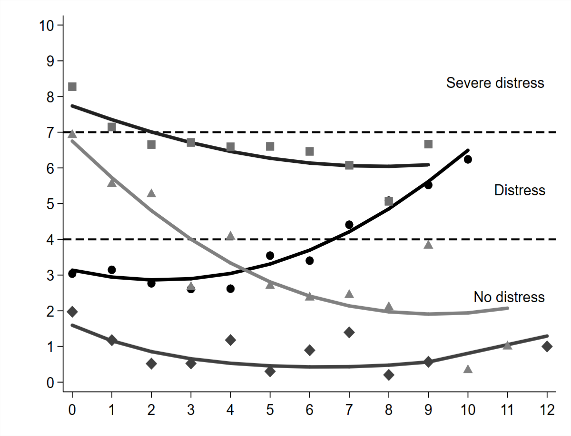


A

B

Distress thermometer

Distress thermometer

Distress thermometer

Distress thermometer

Supplement 3. Summary measures of the number of unmet needs reported by patients in the four different distress trajectory groups.

| **Distress trajectory** | **N** | **mean** | **sd** | **min** | **max** | **median** | **p25** | **p75** |
| --- | --- | --- | --- | --- | --- | --- | --- | --- |
| **1st survey** |  |  |  |  |  |  |  |  |
| Low | 19 | 6.5 | 8.4 | 0 | 34 | 4 | 1 | 8 |
| Low to high | 52 | 17.4 | 13.7 | 0 | 47 | 15 | 5 | 29 |
| High to low | 21 | 24.0 | 13.6 | 0 | 57 | 27 | 15 | 33 |
| High | 24 | 29.5 | 15.6 | 0 | 64 | 26 | 19 | 41 |
|  |  |  |  |  |  |  |  |  |
| Total | 116 | 19.3 | 15.2 | 0 | 64 | 18 | 5 | 31 |
| **2nd survey** |  |  |  |  |  |  |  |  |
| Low | 17 | 11.5 | 15.5 | 0 | 66 | 7 | 3 | 15 |
| Low to high | 36 | 25.8 | 18.9 | 1 | 66 | 22 | 9 | 37 |
| High to low | 19 | 17.7 | 18.0 | 0 | 66 | 13 | 5 | 23 |
| High | 17 | 29.5 | 13.1 | 10 | 52 | 29 | 20 | 39 |
|  |  |  |  |  |  |  |  |  |
| Total | 89 | 22.1 | 18.1 | 0 | 66 | 18 | 7 | 34 |
| **3rd survey** |  |  |  |  |  |  |  |  |
| Low | 13 | 5.8 | 5.7 | 0 | 19 | 6 | 1 | 8 |
| Low to high | 22 | 20.8 | 15.6 | 1 | 66 | 19 | 8 | 30 |
| High to low | 17 | 15.1 | 17.3 | 0 | 66 | 10 | 4 | 16 |
| High | 12 | 24.4 | 15.0 | 3 | 51 | 22 | 13 | 38 |
|  |  |  |  |  |  |  |  |  |
| Total | 64 | 16.9 | 15.6 | 0 | 66 | 11 | 6 | 23 |

Supplement 4. The range of the proportion of patients in each distress trajectory group reporting unmet needs for each of the 66 question in the SNSC survey over three time points 1,2 and 3.

SCNS question number


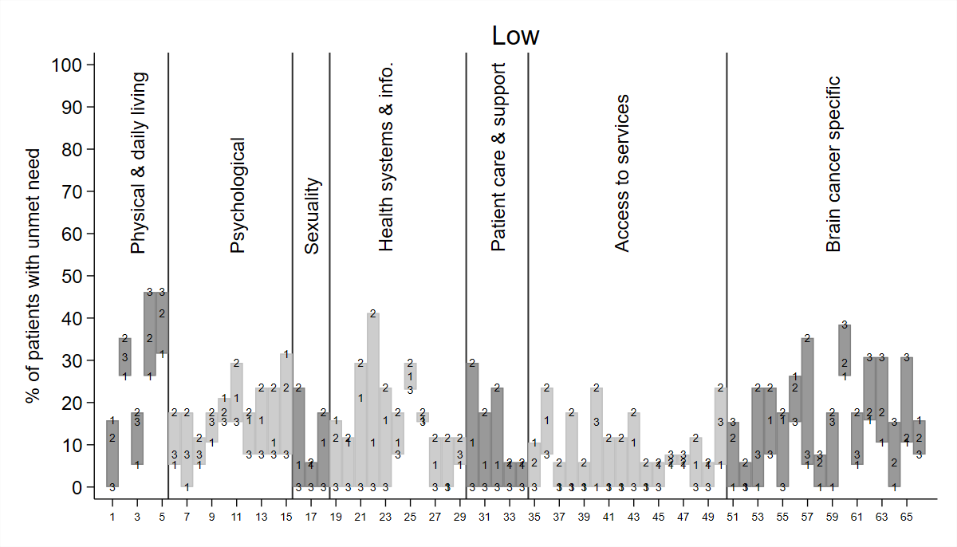

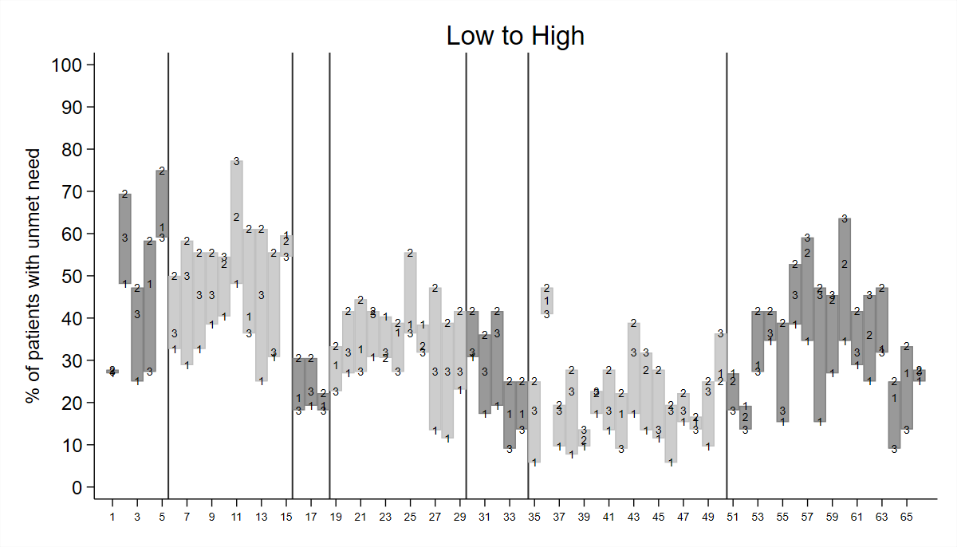

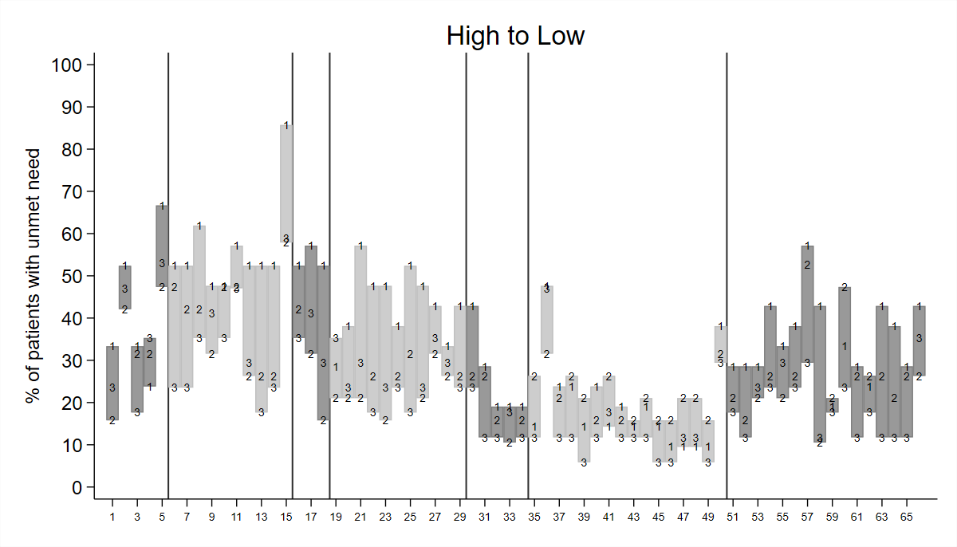

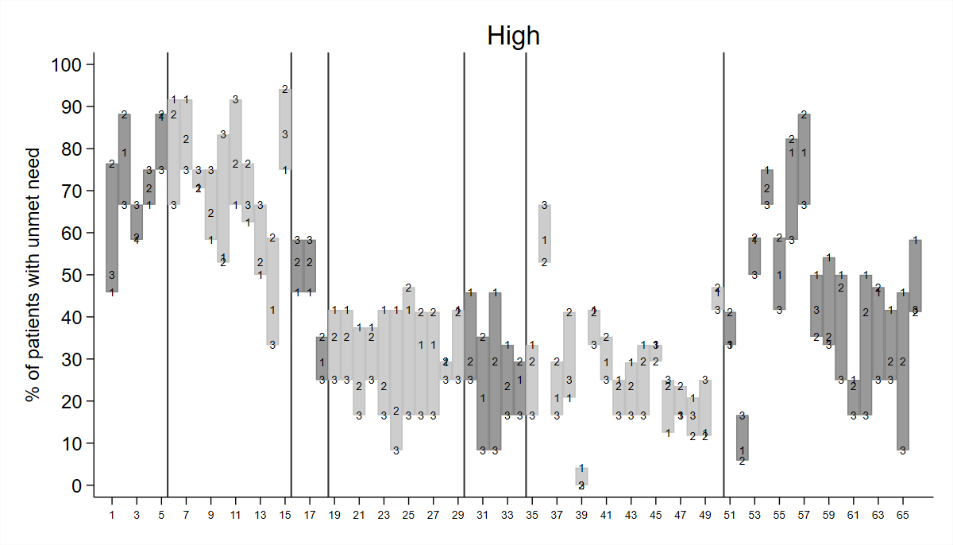


| Key-Unmet needs in each domain |
| --- |
| Psychological   1. Anxiety 2. Feeling down or depressed 3. Feelings of sadness 4. Fears about the cancer spreading 5. Worry that the results of treatment are beyond your control 6. Uncertainty about the future 7. Learning to feel in control of your situation 8. Keeping a positive outlook 9. Feelings about death and dying |
| Sexuality   1. Changes in sexual feelings 2. Changes in your sexual relationships |
| Health systems and info   1. Concerns about the worries of those close to you 2. More choice about which cancer specialists you see 3. More choice about which hospital you attend 4. Reassurance by medical staff that the way you feel is normal 5. Hospital staff attending promptly to your physical needs 6. Hospital staff acknowledging, and showing sensitivity to, your feelings and emotional needs 7. Being given written information about the importance aspects of your care 8. Being given information (written, diagrams, drawings) about aspects of managing your illness and side effects at home 9. Being given explanations of those tests for which you would like explanations 10. Being adequately informed about the benefits and side effects of treatment before you choose to have them 11. Being informed about your test results as soon as feasible 12. Being informed about cancer which is under control or diminishing (that is, remission) 13. Being informed about things you can do to help yourself to get well |
| Patient care and support   1. Having access to professional counselling (eg, psychologist, social worker, counsellor, nurse specialist) if you, family or friends need it 2. To be given information about sexual relationships 3. Being treated like a person, not just another case 4. Being treated in a hospital or clinic that is as physically pleasant as possible 5. Having one member of hospital staff with whom you can talk to about all aspects of your condition, treatment and follow-up |
| Access to services   1. Transport service to and from the hospital or clinic 2. Easy car parking at the hospital or clinic 3. Food and drink facilities in or near the clinic waiting room 4. Comfortable waiting room 5. Childminding at hospital or clinic 6. Counselling services (eg. counsellor, psychologist, social worker, nurse specialist) at the hospital or clinic for your family/partner 7. Brochures about services and benefits for patients with cancer 8. Library of books and videos about cancer and related issues 9. Relaxation classes 10. Drop-in counselling and support service 11. 24-hour telephone support and cancer advisory service 12. Home nursing service 13. Home cleaning service 14. Home gardening service 15. Respite care 16. Monetary allowance for travel, treatment and equipment expenses |
| Brain tumour specific needs   1. Changes in your ability to care for yourself (such as washing and bathing yourself, cooking for yourself 2. Changes in your ability to look after children or other dependents 3. Changes in your appearance 4. Changes in your mental or thinking ability 5. Other people reacting to you differently than before you had the brain tumour 6. Physical side effects from the tumour and/or treatment 7. Feeling like you are not the same person you were before the brain tumour 8. Feeling of being alone in your illness 9. Talking to other people with a similar experience 10. Information on the latest developments in the research and treatment of brain tumours 11. Internet or email to receive information and/or emotional support 12. Assistance with household management ( such as planning ahead, filling out forms, writing letters) 13. Special testing and advice about mental and thinking abilities 14. Legal assistance/advice 15. Rehabilitation services such as occupational therapists, speech pathologists or physiotherapists 16. Financial assistance/advice |
